# Thyroglobulin expression, Ki-67 index, and lymph node ratio in the prognostic assessment of papillary thyroid cancer

**DOI:** 10.1038/s41598-023-27684-3

**Published:** 2023-01-19

**Authors:** Helene Lindfors, Marie Karlsen, Ellinor Karlton, Jan Zedenius, Catharina Larsson, Catharina Ihre Lundgren, C. Christofer Juhlin, Ivan Shabo

**Affiliations:** 1 Department of Surgery, Sundsvall Hospital, 851 86 Sundsvall, Sweden; 2 Department of Molecular Medicine and Surgery, Karolinska Institutet, 171 77 Stockholm, SE Sweden; 3 Department of Breast, Endocrine Tumors, and Sarcoma, Karolinska University Hospital, 171 76 Stockholm, SE Sweden; 4 Department of Oncology-Pathology, Karolinska Institutet, 171 77 Stockholm, SE Sweden; 5 Department of Pathology and Cancer Diagnostics, Karolinska University Hospital, 171 76 Stockholm, SE Sweden

**Keywords:** Thyroid cancer, Surgical oncology

## Abstract

The clinical significance of thyroglobulin (Tg) expression in papillary thyroid cancer (PTC) has not been systematically explored in relation to the Ki-67 index, lymph node ratio (LNR), or other conventional prognostic predictors. In this retrospective study of 327 patients with PTC, we investigated the immunohistochemical expression of Tg in both primary tumors and their matching lymph node metastases in relation to the Ki-67 index, LNR, and clinical data. Tumoral Tg immunoreactivity was inversely correlated to the Ki-67 index and tumor recurrence. The Ki-67 index was higher in lymph node metastases (mean 4%) than in the primary tumors (mean 3%). Reduced Tg expression, estimated as 0–25% Tg positive tumor cells, was more common in lymph node metastases compared to primary tumors. In addition to advanced metastatic burden (defined as N1b stage and LNR ≥ 21%), low Tg expression (0–25% positive tumor cells) in lymph node metastases had a significant prognostic impact with shorter recurrence-free survival. These findings support the potential value of histopathological assessment of Tg expression and Ki-67 index in lymph node metastases as complementary predictors to anticipate the prognosis of PTC patients better.

## Introduction

Papillary thyroid cancer (PTC) is the most common type of differentiated thyroid cancer (DTC) and corresponds to 80–85% of all thyroid malignancies^1^. Despite an excellent prognosis with a 10-year survival rate of 90–95%, PTC exhibits more aggressive behavior in about 20% of cases resulting in increased tumor recurrence and mortality^2^.

At the time of diagnosis, cervical lymph node metastases are found in 30–70% of cases. Lymph node metastasis is one of the most substantial prognostic factors predicting tumor recurrence. Within the first year after primary surgery, recurrent PTC is associated with worse clinical outcomes and increased mortality rates^3,4^.

Thyroglobulin (Tg) is a large glycoprotein that binds iodine and is a precursor in thyroid hormone synthesis. Serum Tg is a highly sensitive and specific marker for tumor recurrence in the surveillance of PTC^5^. Tumor cells in PTC are derived from thyroid follicular cells and are usually highly differentiated, expressing cytoplasmatic Tg. However, Tg expression is often significantly reduced in PTC cells compared with normal adjacent thyroid tissue^6^. It is also suggested that loss or decrease of Tg expression in differentiated thyroid cancer, including PTC, results in impaired functional differentiation of tumor cells with low radioiodine uptake and, hence, decreased response to radioiodine therapy^7–10^. Consequently, radioiodine therapy is generally not recommended in the treatment of PTC lacking the ability to absorb radioiodine^11^.

Currently, immunohistochemical (IHC) detection of Tg is used to diagnose or exclude thyroidal origin in metastases from unknown primary tumors^12^. The clinical impact of Tg IHC in PTC has not been systematically investigated. In a recent study of 49 patients with poorly differentiated thyroid cancer, Walczyk et al. showed that reduced Tg immunoreactivity was an independent risk factor for a worse cancer-specific survival^13^.

This study explores the clinical significance of Tg expression in primary tumors and their matched lymph node metastases in PTC. We hypothesize that a decrease or loss of Tg immunostaining of PTC cells indicates functional dedifferentiation resulting in impaired prognosis and an increased risk of tumor recurrence.

## Material and methods

### Patient material

Patients diagnosed with PTC at the Karolinska University Hospital, Stockholm, Sweden, from 2006 to 2017 were retrospectively included. Altogether all 327 cases that were assessable for Tg expression and Ki-67 analyses were included, whereby no exclusion criteria were applied in this cohort of PTC patients. To obtain a comprehensive evaluation of lymph node metastasis, the inclusion of patients was completed in 2017 when prophylactic central lymph node dissection was terminated as a routine part of thyroid surgery in our department. The study was approved by The Swedish Ethical Review Authority (Reference numbers: 2016/154–32 and 2015/959–31) and performed in accordance with local guidelines and regulations at Karolinska University Hospital. Patients had given their informed biobank consent according to the local guidelines and the Swedish National Law on ethical review of research involving humans. In the case of a preoperatively confirmed cytological diagnosis of PTC, a systematic ultrasound-guided lymph node mapping of all cervical regions was performed by experienced radiologists to explore the presence of pathologically enlarged lymph nodes. Metastasis in pathological lymph nodes was confirmed by fine-needle aspiration and cytological examination.

All patients were treated following the Swedish national guidelines prevailing during the study period, based on ATA^14^ and ETA recommendations^15^. All patients with preoperative PTC diagnosis were treated with total thyroidectomy and prophylactic central cervical lymph node clearance. None of the patients in the study had received chemotherapy after surgery. Patients with preoperatively confirmed lymph node metastases in the lateral cervical compartments during the primary operation were treated with lateral lymph node dissection. In 58 patients, central lymph node dissection was not performed as they did not have a preoperative suspicion of PTC diagnosis (n = 9) or underwent thyroid surgery due to other indications such as thyrotoxicosis (n = 15), follicular neoplasia suspicion (n = 11) or a goiter (n = 23). In addition, 24 patients had preoperatively known PTC, and of these, 20 patients underwent central lymph node dissection, but the number of removed lymph nodes was fewer than 6 nodes, and 4 patients were not treated with central lymph node dissection due to old age, advanced multiple diseases, or distant metastasized advanced PTC. Hence, 82 patients (25%) had Nx tumors due to waived lymph node dissection or detected central lymph nodes of less than 6 nodes. All patients with Nx tumors were excluded from further statistical analysis regarding the lymph node ratio (LNR) calculation as well as analysis of Tg and Ki-67 expression in LN metastases.

### Histopathologic examination

The primary tumor and lymph node metastasis data were obtained from pathology reports following the primary surgery. Histopathological diagnostics were performed according to routine clinical practice by endocrine pathologists at the Department of Pathology and Cancer Diagnostics, Karolinska University Hospital, Stockholm, Sweden, following the current WHO classification during the study period. The following data were collected: number and size of the primary PTCs, presence of extrathyroidal extension, presence of thyroiditis, the total number of observed lymph nodes, the number of lymph nodes with metastasis in all cervical regions, and the status of the resection margins. Cases presenting extrathyroidal extension were subsequently subclassified as microscopic or gross. Gross tumor extension was defined by the surgeons' judgement in the medical record, including operation charts, and not the histopathological report. Lymph node metastases were subclassified as macro- or micro metastases, where micro metastases were defined as clusters of tumor cells < 2.0 mm in diameter.

Immunostaining of the primary tumors and lymph node metastases was performed according to clinical laboratory standards. When assessed, the primary thyroid tumor and the largest lymph node metastases were analyzed for Tg and Ki-67 immunohistochemistry at our pathology department. During the study period, the samples were stained for Tg using an automated methodology with an immunohistochemical staining machine. The equipment used from 2006 to Sept 2015 was a Bond MAX immuno platform (Leica Biosystems) and, from Oct 2015, the Ventana BenchMark ULTRA stainer. The Tg antibody used with the Bond machine was a rabbit polyclonal antibody (product number 0251, Agilent, with a working dilution of 1:10.000 with antigen retrieval heat pre-treatment using citrate buffer according to the manufacturer’s instructions. The Tg antibody used with the Ventana Benchmark Ultra was a mouse monoclonal antibody (clone 2H11 + 6E1), used at 0,93 μg/ml) with a similar heat pretreatment as for the Bond machine. To ensure the quality of the staining for Tg in clinical routine, normal thyroid tissue was used as an internal control; for negative controls, different tissues were used, such as kidney, lymph node and colon. For Ki-67, two different automated staining procedures were used during the study period. First, the Ventana BenchMark XT was used until 2009, followed by the Ventana BenchMark ULTRA stainer until the end of the study period. Then, the Mib-1 antibody clone (Immunotech, Marseille, France) was used as a primary antibody until 2016, when the CONFIRM anti-Ki-67 antibody (clone 30–9, Roche, Basel, Switzerland) was used, with lymph nodes as positive controls and various tissues from colon, pancreas, and kidney as negative controls.

**Figure 1:**
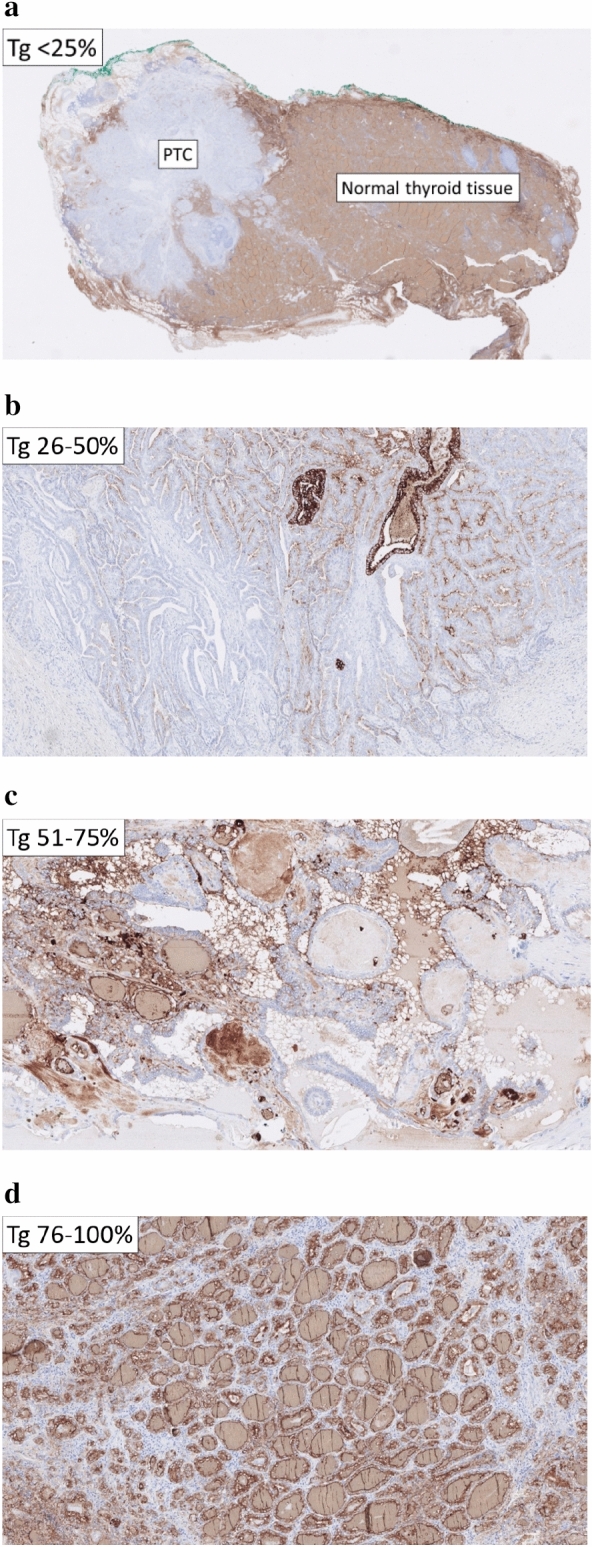
Histological images representative of each semi-quantitively graded group of thyroglobulin immunostaining confined to tumor cells in papillary thyroid cancer (**a**) 0–25%, (**b**) 26–50%, (**c**) 51–75%, and (**d**) 76–100% stained tumor cells.

The Ki-67 labeling index was calculated by manually counting the Ki-67 positive tumor cell nuclei divided by the total amount of tumor cell nuclei in “hot-spot” regions, counting at least 2000 cells using an ocular grid. The Tg expression in tumor cells (cytoplasmatic staining) was estimated semi-quantitatively and assigned to the following categories: 0–25%, 25–50%, 50–75%, and 75–100% of tumor cells expressing Tg (Fig. 1). The LNR was defined as the total number of metastatic lymph nodes divided by the total number of lymph nodes retrieved from the central and lateral cervical compartments.

### Clinical follow-up

Six to eight weeks after primary surgery, the patients were offered radioiodine treatment and post-irradiation whole-body scintigraphy. The first regular follow-up control was 9–12 months after completing the primary treatment and consisted of physical examination and blood tests, including the level of TSH suppression and serum Tg. To obtain a uniform analysis of tumor recurrence independent of radioiodine treatment, all patients who did not receive radioiodine treatment postoperatively were excluded from all analysis with tumor recurrence as primary endpoint. The aim was not to evaluate the outcome of radioiodine treatment but to predict relapse after complete routine treatment of PTC. In the case of recurrence-free outcomes, the subsequent controls continued annually. Tumor recurrence was defined as any of the following events that occurred at least six months after radioiodine therapy: a biochemical sign of recurrence (increase of serum Tg), radiological and/or cytological confirmation of regional lymph node, or distant metastasis.

### Statistical analysis

All statistical analyses were performed using SPSS statistics software version 26 (IBM Inc., Chicago, USA). Pearson's *χ*^2^ test was used to assess the difference in the distribution of a categorical clinical variable regarding the presence of lymph node metastasis and tumor recurrence. To evaluate the performance of means for LNR and Ki-67 expression rates in the primary tumor and lymph node metastases as cut-off values, we used receiver operating characteristic (ROC) analysis. The sensitivity and 1-minus specificity data over tumor recurrence-free survival as outcomes were used. The area under the curve (AUC) was calculated with 95% CIs for LNR and rates of Ki-67 means as a dichotomous variable.

Comparison of clinical data, expression of Tg (4 categories), and Ki-67 index were achieved by one-way analysis of variance (ANOVA) with post-hoc testing and Bonferroni correction. Differences in the Ki-67 index between the primary tumor and their matched lymph node metastases were assessed by Spearman´s rank order correlation test. Pearson's χ^2^ test and Cohen test were used to evaluate correlation agreement in the distribution of Tg expression between the primary tumors and their corresponding lymph node metastases. The distribution of Tg expression and Ki-67 index between these two sites was illustrated by G-graph and heat map. Survival rates, estimated according to Kaplan–Meier curves with multiple comparison corrections, were based on recurrence-free survival (RFS) and calculated in relation to Tg and Ki-67 expression. Log-rank tests were used to analyze the differences between survival rates and were considered significant at *p* < 0.05.

## Results

The study includes 327 patients, of which 245 (75%) are female and have a median age of 44 years (range 10–85) at the time of diagnosis. The mean follow-up time was 103 months (range 2 to 176 months). After surgery, 2 patients (1%) had the gross residual disease (R2), 52 (16%) had a microscopic positive margin of resection (R1), and 273 (83%) had complete resection with negative margins (R0). The primary tumors were multifocal in 142 (43%). The tumor size was > 30 mm in 80 (24%) patients. Extrathyroidal extension was found in 110 (34%) patients; out of those, one patient had gross tumor extension. Lymph node metastasis was found in 190 (58%) patients; out of those, 31 (16%) patients had micro metastasis. Tumor recurrence occurred in 40 (12%) patients.

**Figure 2:**
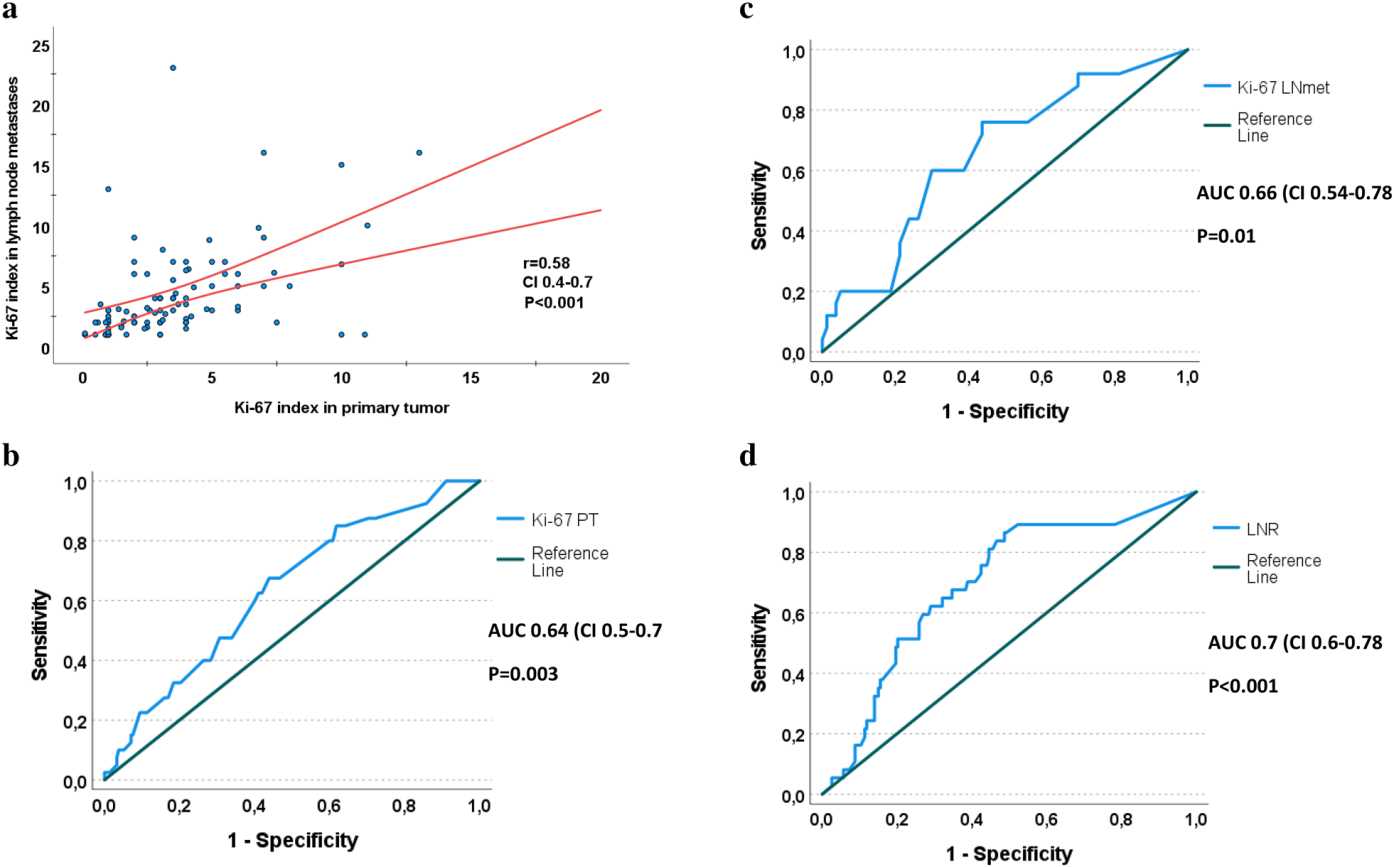

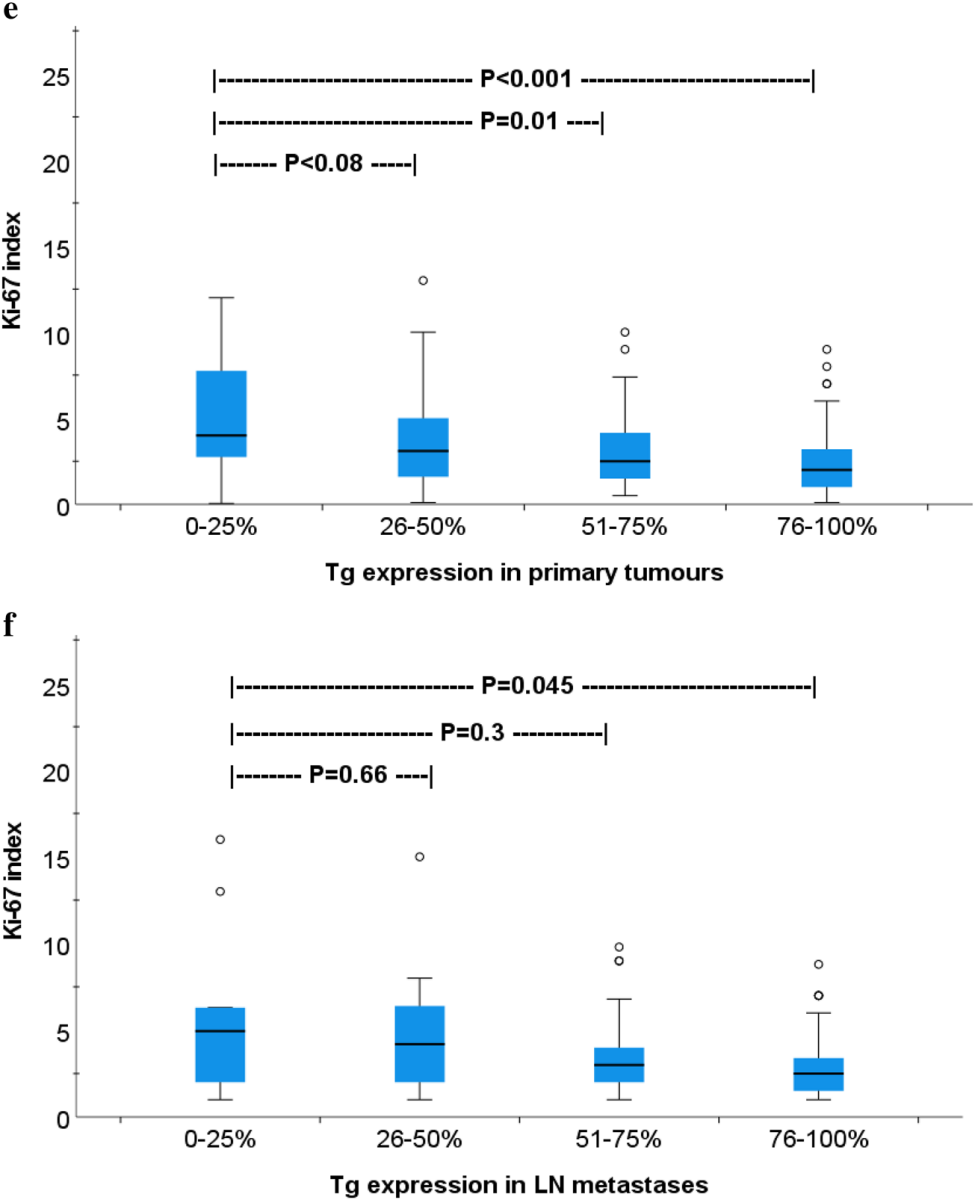
The relationship between Ki-67 labeling index and thyroglobulin expression in papillary thyroid cancer. (**a**) Scatter plot showing the relationship between the Ki-67 index in the primary tumors and their matching lymph node metastases. Receiver operating characteristic (ROC) curves illustrating the ability of the Ki-67 index in (**b**) primary tumors (PT) and (**c**) lymph node metastases (LNmet), and (**d**) lymph node ratio (LNR) in predicting tumor recurrence of papillary thyroid cancer. The highest AUC identified for Ki-67 ≥ 2.45% in primary tumors was 0.64, with a sensitivity of 68% and specificity of 56% (CI 0.5–0.7, *p* = 0.003). For the Ki-67 index in lymph node metastases, a labeling index of ≥ 2.85% was predictive for tumor recurrences with a sensitivity of 76%, specificity of 52%, and AUC of 0.66 (CI 0.54–0.78, *p* = 0.01). Lymph node ratio ≥ 21% was predictive for tumor recurrence with a sensitivity of 81%, specificity of 60%, and AUC 0.7 (CI 0.6–0.78, *p* < 0.001). ANOVA analysis evaluating the correlation between thyroglobulin expression and Ki-67 index in primary PTC (**e**) and their paired lymph node metastases (**f**).

The mean Ki-67 index in the primary tumor was 3%, and in the lymph node metastases was 4%. Ki-67 index in primary tumors correlates positively with that in lymph node metastases (r = 0.58, CI 0.4–0.7 and *p* < 0.001) (Fig. 2a). Based on ROC analysis and in relation to tumor recurrence, the highest AUC identified for Ki-67 ≥ 2.45% in primary tumors was 0.64 with a sensitivity of 68% and specificity of 56% (CI 0.5–0.7, *p* = 0.003) (Fig. 2b). For the Ki-67 index in lymph node metastases, a labeling index of ≥ 2.85% was predictive for tumor recurrences with a sensitivity of 76%, specificity of 52%, and AUC of 0.66 (CI 0.54–0.78, *p* = 0.01) (Fig. 2c). Since the biological behavior of cancer cells is believed to differ in primary tumors compared to metastases^16,17^, different cut-off rates of the Ki-67 index for these two sites were used in the subsequent statistical comparison. Sixty (18%) patients did not receive radioiodine treatment as they had microcarcinoma or were older with multiple diseases and were excluded from statistical comparisons in relation to tumor recurrence. More details about patient characteristics are specified in Table 1.

**Table 1:** Patient characteristics.

	Patient N (%)
**Gender**	
Male	82 (25)
Female	245 (75)
**Age group**	
< 40 years	54 (16)
40–49 years	84 (26)
50–59 years	68 (21)
60–69 years	49 (15)
≥ 70 years	72 (22)
**Tumor size** (mm)	
No evidence of primary	1 (0.3)
≤ 10	76 (23)
11–20	111 (34)
21–30	59 (18)
31–40	33 (10)
> 40	47 (14)
**N-stage**	
N0	55 (17)
N1a	108 (33)
N1b	82 (25)
Nx	82 (25)
**Lymph node ratio (%)**	
< 21	125 (51)
≥ 21	119 (49)
**Thyroiditis**	
No	233 (71)
Yes	94 (29)
**Tumor focality**	
Unifocal	184 (56.3)
Multifocal	142 (43.4)
Tx	1 (0.3)
**Complete resection**	
R0	273 (83)
R1	52 (16)
R2	2 (1)
**Extrathyroidal extension**	
No	217 (66)
Yes	110 (34)
**Thyroglobulin expression in primary tumor (%)**	
0–25%	20 (6)
26–50%	40 (12)
51–75%	55 (17)
76–100%	153 (47)
Missing data	59 (18)
**Thyroglobulin expression in lymph node metastases (%)**	
0–25%	15 (4.6)
26–50%	15 (4.6)
51–75%	21 (6.4)
76–100%	55 (17)
Missing data	221 (68)
**Ki-67 index primary tumor (%)**	
< 2.45%	158 (48)
≥ 2.45%	131 (40)
Missing data	38 (12)
**Ki-67 index lymph node metastases (%)**	
< 2.85%	51 (16)
≥ 2.85%	54 (16)
Missing data	222 (68)

### The presence of lymph node metastases

The presence of lymph node metastasis is related to extrathyroidal extension and multifocal tumors. Out of 101 patients with extrathyroidal extension, 87 (86%) had lymph node metastasis at the time of surgery (*p* < 0.001). The corresponding rates for patients with multifocal tumors were 97 out of 125 patients (78%) (*p* = 0.018). No relation was found between the presence of lymph node metastasis and other clinical variables such as age, tumor size, thyroiditis, Ki-67 index, or Tg expression in primary tumors (Table 2). Based on ROC analysis, lymph node ratio ≥ 21% was predictive for tumor recurrence with a sensitivity of 81%, specificity of 60%, and AUC 0.7 (CI 0.6–0.78, *p* < 0.001) (Fig. 2d).

**Table 2:** Univariate analysis comparing the presence of cervical lymph node metastasis at the time of diagnosis in relation to clinicopathological data of papillary thyroid cancer patients.

	Lymph node metastasis		
	No N (%)	Yes N (%)	*p*
**Gender**			
Female	59 (75)	136 (72)	0.6
Male	20 (25)	54 (28)	
**Age group**			
< 40 years	10 (13)	39 (20)	0.17
40–49 years	18 (23)	56 (30)	
50–59 years	16 (20)	39 (20)	
60–69 years	13 (16)	22 (12)	
≥ 70 years	22 (28)	34 (18)	
**Tumor size**			
≤ 10 mm	6 (7.6)	38 (20)	0.05
11–20 mm	36 (45.6)	63 (33.2)	
21–30 mm	16 (20.3)	36 (18.9)	
31–40 mm	12 (15.2)	21 (11.1)	
> 40 mm	9 (11.4)	32 (16.8)	
**Thyroiditis**			
No	55 (70)	130 (68)	0.8
Yes	24 (30)	60 (32)	
**Tumor focality**			
Unifocal	51 (65)	92 (49)	0.018
Multifocal	28 (35)	97 (51)	
**Extrathyroidal extension**			
No	65 (82)	103 (54)	< 0.001
Yes	14 (18)	87 (46)	
**Ki-67 index in primary tumor (%)**			
< 2.45	44 (60)	84 (47)	0.055
≥ 2.45	29 (40)	95 (53)	
**Thyroglobulin expression in primary tumor (%)**			
0–25	5 (7)	13 (7)	0.9
26–50	9 (13)	30 (17)	
51–75	14 (20)	36 (21)	
76–100	42 (60)	97 (55)	

### Tumor recurrence

Tumor recurrence was not found in patients with primary tumors ≤ 10 mm, but it was more common in patients with tumors > 30 mm (17 out of 74 cases, 23%) compared to having 11–30 mm tumors (23 out of 155 cases 15%) (*p* = 0.008). The tumor relapse was related to advanced N stage and metastatic burden estimated as LNR ≥ 21%. Tumor recurrence was more frequent in patients with the N1b stage (25 out of 81 cases, 31%) compared to N0 (4 out of 47, 8.5%) and N1a (8 out of 103, 8%) stages (*p* < 0.001). Of 117 cases, 30 patients (26%) with LNR ≥ 21% experienced tumor recurrence. The corresponding rate in patients with LNR < 21% was 7 out of 113 patients (6%) (*p* < 0.001) (Table 3).

**Table 3:** Univariate analysis comparing tumor recurrence in relation to clinicopathological data of papillary thyroid cancer patients.

	Tumor recurrence		
	No N (%)	Yes N (%)	*p*
**Gender**			
Female	167 (74)	27 (68)	0.4
Male	60 (26)	13 (32)	
**Age group**			
< 40 years	37 (16.3)	13 (32.5)	0.01
40–49 years	61 (26.9)	9 (22.5)	
50–59 years	50 (22)	5 (12.5)	
60–69 years	37 (16.3)	1 (2.5)	
≥ 70 years	42 (18.5)	12 (30)	
**Tumor size**			
≤ 10 mm	38 (16.7)	0 (0)	0.008
11–20 mm	88 (38.8)	11 (27.5)	
21–30 mm	44 (19.4)	12 (30)	
31–40 mm	23 (10.1)	8 (20)	
> 40 mm	34 (15)	9 (22.5)	
**N-stage**			
N0	43 (18.9)	4 (10)	< 0.001
N1a	95 (41.9)	8 (20)	
N1b	56 (24.7)	25 (62.5)	
Nx	33 (14.5)	3 (7.5)	
**Lymph node ratio (%)**			
< 21	106 (55)	7 (19)	< 0.001
≥ 21	87 (45)	30 (81)	
**Radical resection**			
No	36 (16)	14 (35)	0.004
Yes	191 (84)	26 (65)	
**Thyroiditis**			
No	155 (68)	31 (78)	0.24
Yes	72 (32)	9 (22)	
**Tumor focality**			
Unifocal	124 (55)	16 (40)	0.09
Multifocal	102 (45)	24 (60)	
**Extrathyroidal extension**			
No	150 (66)	15 (38)	< 0.001
Yes	77 (34)	25 (62)	
**Ki-67 index i**n **primary tumor (%)**			
< 2.45	145 (58.5)	13 (32)	0.001
≥ 2.45	103 (41.5)	28 (68)	
**Ki-67 index in lymph node metastases (%)**			
< 2.85	45 (56)	6 (24)	0.005
≥ 2.85	35 (44)	19 (76)	
**Thyroglobulin expression in primary tumor (%)**			
0–25	11 (5.4)	6 (15.4)	0.025
26–50	30 (14.6)	9 (23.1)	
51–75	39 (19)	9 (23.1)	
76–100	125 (61)	15 (38.5)	
**Thyroglobulin expression in lymph node metastases (%)**			
0–25	10 (13)	5 (19)	0.002
26–50	8 (10)	7 (27)	
51–75	12 (15)	9 (35)	
76–100	49 (62)	5 (19)	

Tumor recurrence was significantly associated with increased Ki-67 index and decreased Tg expression in both primary tumor and lymph node metastasis. Tumor relapse was more common in patients having tumors with Ki-67 index ≥ 2.45% (28 out of 131, 21%, *p* = 0.001) and lymph node metastases with Ki-67 ≥ 2.85% (19 out of 54, 35%) (*p* = 0.005). The corresponding rates for patients having tumors with Ki-67 index < 2.45% and lymph node metastases with Ki-67 < 2.85% were 13 out of 158 patients (8%) and 6 out of 51 patients (12%), respectively. Tumor recurrence was inversely correlated to Tg expression as it was found in 6 out of 17 (35%), 9 out of 39 (23%), 9 out of 48 (18%), and 15 out of 140 (10%) of patients with tumors expressing Tg in 0–25%, 26–50%, 51–75% and 76–100% of tumors cells (*p* = 0.025), respectively. The corresponding rates of tumor recurrence in relation to Tg expression in lymph node metastases were 5 out of 15 (33%), 7 out of 15 (46%), 9 out of 21 (42%), and 5 out of 54 (9%) patients (*p* = 0.002) (Table 3).

In addition, recurrence was significantly related to the age of the patients (*p* = 0.01), extrathyroidal extension (*p* < 0.001), tumor radicality (*p* = 0.004), and tumor size (*p* = 0.008) but not associated with patient gender, the presence of thyroiditis nor number of primary tumors (Table 3).

### The Ki-67 index is inversely related to the Tg expression

Ki-67 index in primary tumors and their corresponding lymph node metastasis was inversely correlated to Tg expression. The mean Ki-67 index in primary tumors expressing Tg 0–25% was 5.3%, which is significantly higher compared to tumors with Tg expression of 51–75% (*p* = 0.01) and 76–100% (*p* < 0.001) (Fig. 2e).

The mean Ki-67 index in lymph node metastases with Tg expression of 0–25% (6.6%) was higher compared to the other Tg expression categories, 26–50% (4.7%), 52–75% (3.9%), and 67–100% (3%), but only statistically significant in relation to metastases expressing Tg in 76–100% of tumor cells (*p* = 0.045) (Fig. 2f).

**Figure 3:**
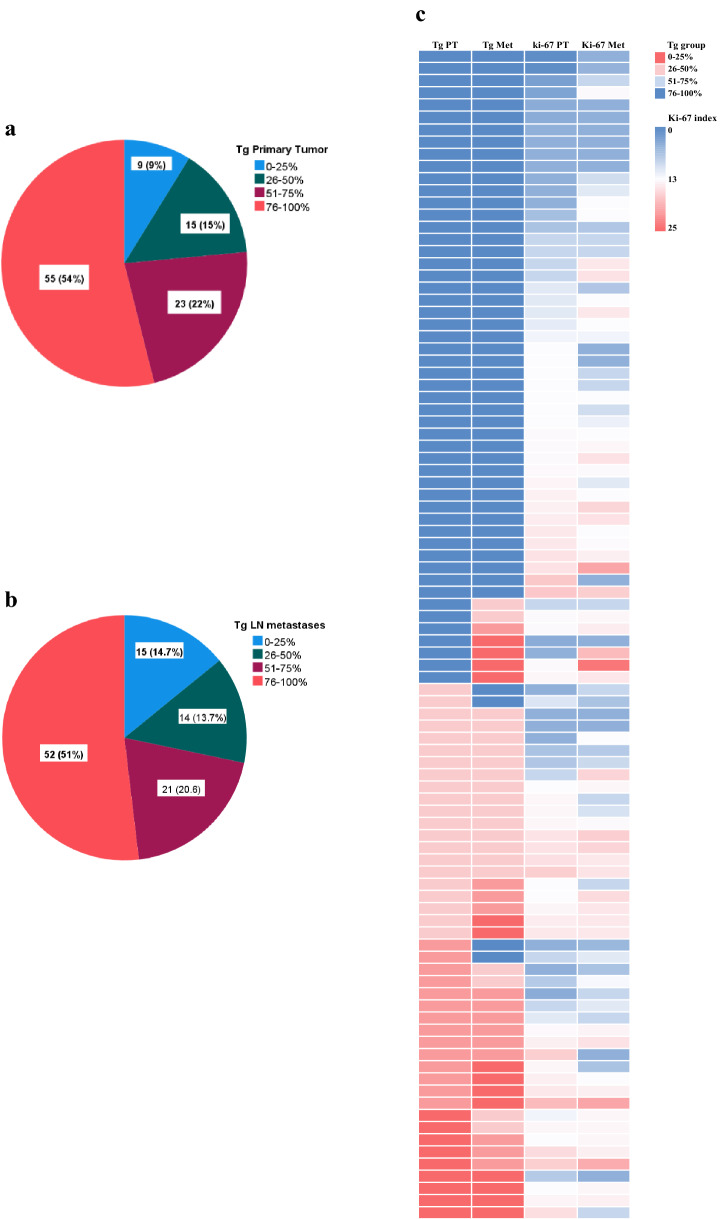
*G*-graph (**a** and **b**) and heat map (**c**) illustrate the distribution of thyroglobulin expression in primary papillary thyroid cancer and their paired lymph node metastases.

Out of 327 patients, data regarding Tg expression in primary tumors was found in 268 (82%) and matched lymph node metastases in 105 (32%) cases. In primary tumors, the distribution of Tg expression rates were 0–25%; n = 20 (7.5%), 26–50%; n = 40 (15%), 51–75%; n = 55 (20.5%) and 76–100%; n = 153 (57%) patients. The corresponding rates for Tg expression in lymph node metastases were n = 15 (14%), n = 15 (14%), n = 21 (20%), and n = 55 (52%) (Fig. 3a,b). To explore the consistency of Tg expression between the primary tumor and its matching lymph node metastases (102 patients), we measured the correlation and agreement between these two sites. Spearman correlation showed a moderate correlation (R = 0.63) with a fair agreement (Kappa = 0.54) between the Tg expression in primary tumors and their corresponding lymph node metastases. The proportion of metastases that continued to have the same level of Tg expression as in the primary tumor was higher in tumors with Tg 76–100%; n = 47 (85.5%) compared to the other Tg groups; 0–25%, n = 4 (44%), 26–50%; n = 7 (47%) and 51–75%; n = 14 (61%). These differences in Ki-67 index and Tg expression between the primary tumors and their matching lymph node metastases are visualized in a heat map in Fig. 3c.

### Recurrence-free survival in relation to Tg expression

**Figure 4:**
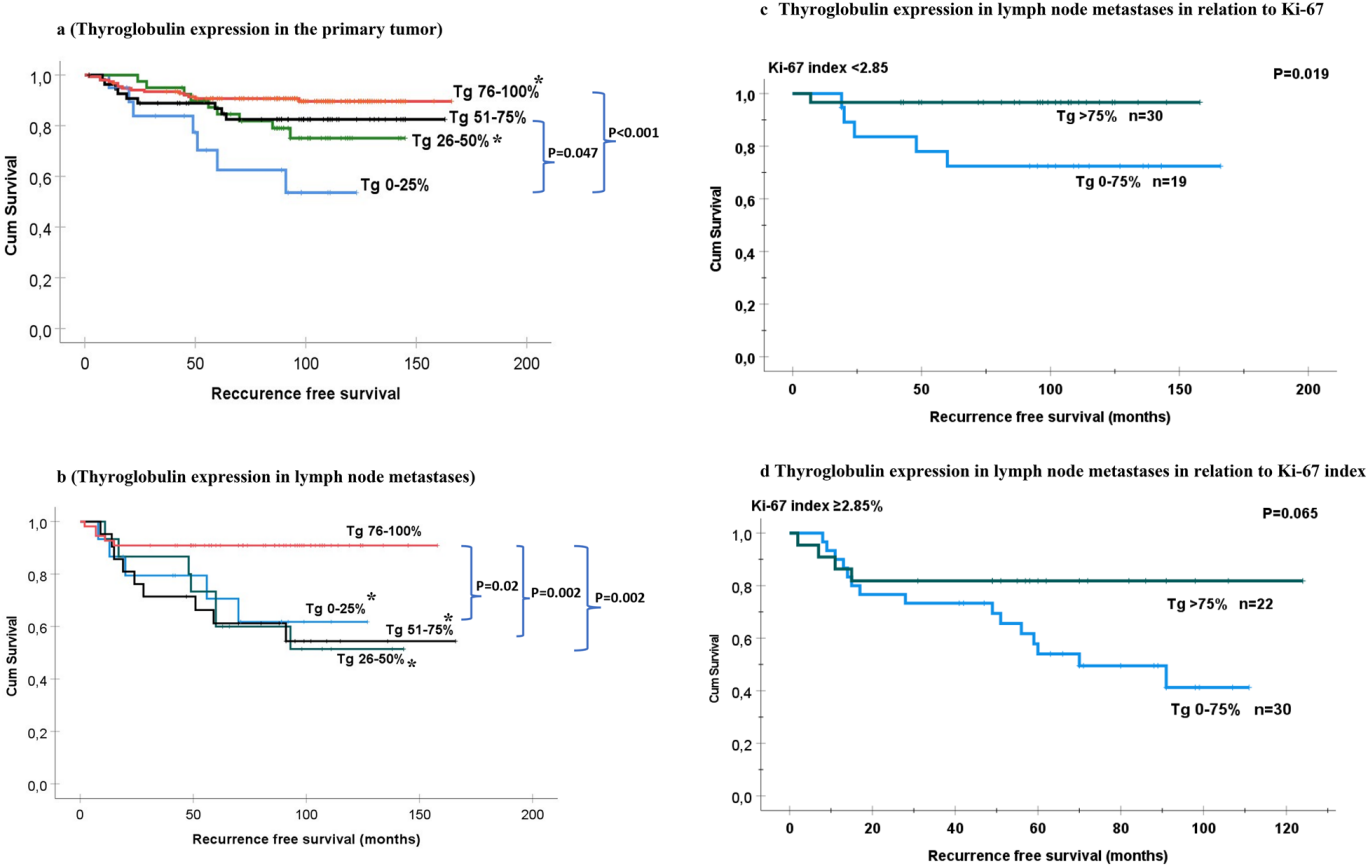
Recurrence-free survival (RFS) in patients with papillary thyroid cancer was analyzed as Kaplan–Meier curves with multiple comparison corrections comparing tumor recurrence in relation to thyroglobulin expression in primary PTC (**a**) and paired lymph node metastases (**b**). In panels (**c**) and (**d**), RFS is estimated in relation to thyroglobulin expression and Ki-67 index in PTC lymph node metastases.

The recurrence-free time was inversely correlated to Tg expression in primary tumors and lymph metastases. Patients with primary PTC exhibiting Tg expression of 0–25% had a recurrence-free time of 88 months, which was significantly shorter compared to those who had tumors with Tg 51–75% (RFS 140 months, *p* = 0.047) and Tg 76–100% (RFS 152 months, *p* < 0.001). Patients with primary tumors expressing Tg 26–50% had RFS of 124 months, which was significantly shorter only compared to patients having PTC with Tg expression 76–100%, but no statistical difference in RFS was found compared to patients with other Tg expression groups (Fig. 4a).

Patients having lymph node metastases with Tg 76–100% had RFS of 144 months, which was significantly longer than for patients with metastases expressing Tg 51–75% (107 months, *p* = 0.002), 26–50% (98 months, *p* = 0.002) and 0–25% (*p* = 0.02). There was no statistical difference in RFS among patients in Tg groups 0–75% (Fig. 4b). Thus, we found differences in RFS if the primary tumor expressed Tg of more or less than 50% and if the metastases had Tg expression of more or less than 75%.

### The prognostic relevance of Tg expression

Multivariate Cox’s proportional hazards model was used to explore the prognostic impact of Ki-67 index and Tg expression in primary PTC and their corresponding lymph node metastases adjusted to other known PTC predictors, such as tumor size and N stage, extrathyroidal extension, and age.

Lymph node ratio ≥ 21%, N1b stage, and Tg expression 0–25% in lymph node metastases were associated with shorter RFS with HR rates of 15 (*p* = 0.023), 14 (*p* = 0.004) and 13.7 (*p* = 0.03), respectively. The other clinical predictors, such as age, tumor size, R0 resection, extrathyroidal extension, Ki-67 (neither in primary tumor nor metastases), and Tg expression in the primary tumor, did not influence the prognosis (Table 4).

**Table 4:** Multivariate Cox proportional hazards model comparing tumor recurrence in relation to clinicopathological data in papillary thyroid cancer patients.

	Hazard ratio (CI)	*p*
**Age group**		
< 40 years	1	
40–49 years	0.001 (0–165)	0.27
50–59 years	1.9 (0.5–6.7)	0.3
60–69 years	2 (0.45–9)	0.35
≥ 70 years	2.5 (0.4–15)	0.2
**Tumor size**		
≤ 10 mm	1	
11–20 mm	1.5 (0.36–6.5)	0.56
21–30 mm	4 (0.77–23)	0.1
31–40 mm	0.02 (0–50)	0.4
> 40 mm	1.7 (0.46–6)	0.4
**N-stage**		
N1a	1	
N1b	14 (2.3–84.8)	0.004
**Lymph node ratio (%)**		
< 21	1	
≥ 21	15 (1.4–154)	0.023
**Radical resection**		
No	1	
Yes	0.8 (0.28–2.3)	0.7
**Extrathyroidal extension**		
No	1	
Yes	3 (0.96–8.6)	0.06
**Ki-67 index in primary tumor (%)**		
< 2.45	1	
≥ 2.45	0.5 (0.1–2.0)	0.33
**Ki-67 index in lymph node metastases (%)**		
< 2.85	1	
≥ 2.85	3 (0.8–11)	0.1
**Thyroglobulin in primary tumor (%)**		
76–100	1	
51–75	0.5 (0.06–4.3)	0.55
26–50	0.21 (0.03–2.1)	0.21
0–25	0.4 (0.07–2.7)	0.36
**Thyroglobulin in lymph node metastases (%)**		
76–100	1	
51–75	4.6 (0.6–36)	0.15
26–50	9 (1–91)	0.055
0–25	13 (1.2–133)	0.03

### Subgroup analysis: prognostic value of Ki-67 index and Tg expression in lymph node metastases

Since low Tg expression in lymph node metastases was significantly related to shorter RFS and increased Ki-67 index, we explored the relevance of the simultaneous expression of Tg expression and Ki-67 index in metastases. In this subgroup analysis, we used a Tg expression cut-off rate of 75% as there was no difference in RFS between patients with lymph node metastases in which Tg expression was 26–50%, 51–75%, or 76–100%.

For patients who had lymph node metastases with Ki-67 index < 2.85%, Tg expression > 75% was associated with longer RFS (153 months) compared to those having lymph node metastases expressing Tg 0–75% (130 months) (*p* = 0.019). No differences in RFS in relation to Tg expression were found among patients whose metastases had Ki-67 ≥ 2.85% (Fig. 4c,d).

## Discussion

The clinical significance of Tg expression in the primary tumor and its matching lymph node metastases in PTC has not been systematically studied in large consecutive series. In the present retrospective study, immunostaining of Tg in primary tumors and lymph node metastases were examined in relation to the Ki-67 index, lymph node ratio, and other conventional prognostic variables in 327 patients with PTC. The expression of Tg was inversely correlated to the Ki-67 index and related to tumor recurrence but not to the presence of lymph node metastasis. In addition to advanced N-stage (N1b, metastasis to the lateral compartment), reduced Tg expression (0–25% positivity) in metastatic lymph nodes and LNR ≥ 21% had a significant prognostic impact, constituting independent clinical predictors associated with shorter recurrence-free survival.

The de-differentiation process is stepwise, resulting in tumor cells with metastatic phenotype and progression of PTC to poorly differentiated and finally anaplastic thyroid cancer^18,19^. Comprehensive experimental^20–22^ and clinical^23,24^ data indicate that the de-differentiation of PTC is associated with accelerated growth, characterized by increased Ki-67 labeling index and a decrease in expression of thyroid differentiation markers such as Tg and TTF-1. Consistent with these observations, we found that Tg expression was inversely correlated to Ki-67 expression in primary PTC and lymph node metastases. The tumor cells in lymph node metastases had a higher Ki-67 index and, to a greater extent, exhibited less Tg expression than the primary tumor, indicating a trend toward functional de-differentiation of PTC cells during the metastatic process. De-differentiation is a key feature of cancer progression^25^. Phenotypic differences, including tissue-specific markers, between metastatic tumor cells and those in the primary site are observed in several solid tumors such as breast, pulmonary, and bladder cancers^26–28^. These differences are likely due to genetic alternations in tumor cells during the metastatic process, but they can also be explained by differences in the microenvironment between these sites^29–32^.

For PTC, however, the differences in the phenotypic and genetic profile of the primary tumor and its metastases are addressed in a few studies only. Masoodi et al.^33^ reported that mutations in PTC driver genes, such as BRAF, NRAS, and HRAS, were shared in primary tumors and metastases but occurred at significantly higher rates in metastatic PTC tissues. In a similar study, Cañadas-Garre et al.^34^ investigated the genetic differences between primary tumors, lymph node metastases, and samples from recurrent disease. The BRAF mutations were heterogeneously distributed among these three sites^33,34^. Melo et al. performed a mutational analysis of DTC metastases (lymph node and distant metastases) and their paired primary tumor from 204 patients, mainly with PTC (n = 180). The authors found genetic concordance between primary PTC and respective lymph node metastases. However, they observed a significant enrichment in TERT promoter mutations (12.9% in primary tumor *vs.* 5.3% in metastases) and decreased frequency of BRAF mutations (44.6% in primary tumor vs. 2.3.8% in metastases) in distant metastasis compared to their paired primary tumor^35^. These data indicate that the primary PTC and paired metastases differ in their range of somatic and phenotypic alterations providing a new source of potential clinical predictors that might influence decisions for personalized therapy in PTC patients with distant metastasis.

Thyroglobulin is produced by thyroid follicular cells only and is detected in patients with remanent normal thyroid tissue or recurrent differentiated thyroid cancer^5^. The level of serum Tg is associated with the amount of remanent DTC or normal thyroid tissue^6^. Hence, radioiodine uptake and, subsequently, its therapeutic effect depends on cytoplasmic Tg expression in tumor cells and their ability to concentrate and retain iodine^36,37^. Recently, Nilsson et al. could show that radioiodine uptake in both PTC, and corresponding lymph node metastases was positively related to Tg expression in tumor cells and that lower radioiodine avidity was more common in metastases compared to primary PTC^38^. These observations indicate that estimation of histopathological Tg expression in PTC tumor tissue may be indicative of predicting radioiodine uptake and, thus, expected therapeutic impact.

Radioiodine therapy is offered after thyroid surgery^5,39^, and since the primary tumor is already removed, radioiodine treatment in the adjuvant setting is expected to act on only metastatic residual tissue. Thus, it is logical to explore the effect of radioiodine treatment on biological properties and prognostic biomarkers of PTC in metastatic lesions rather than in the primary tumor. This concept is consistent with the previous notion in tumor biology that oncological treatment should be targeted against cancer cells in tumor metastases or subpopulations of metastatic cancer cells rather than those in primary tumors^40–43^. In this study, the Tg expression in lymph node metastases outperformed Tg expression in primary PTC as a prognostic indicator. Moreover, we found that patients with lymph node metastases with increased proliferation (Ki-67 ≥ 2.85%) had no difference in RFS regardless of Tg expression. Hypothetically, these findings indicate that tumor cells with accelerated growth might retain radioiodine avidity but might be radioresistant and survive the radiation effect of radioiodine, developing dormant cells that later might result in tumor recurrence.

Although the risk stratification of PTC after complete initial treatment includes several other parameters such as age at diagnosis, histologic features, and extrathyroidal extension, the N-stage has superior prognostic significance^14,44^. The current N-stage classification in PTC is based on anatomical location without considering the extent of lymph node metastases. An increasing number of metastatic nodes is proportional to tumor burden and associated with an increased risk of tumor recurrence and mortality^45–47^. In a retrospective study including 165 PTC patients, we could recently show that LNR ≥ 21% was associated with tumor recurrence regardless of the anatomical site of cervical lymph node metastases. The Ki-67 proliferation index correlated to increased lymph node ratio as tumors in patients with LNR ≥ 21% exhibited a significantly higher Ki-67 index than those with LNR < 21%^48^. In the current study, the patient cohort is extended. The prognostic impact of lymph node ratio is examined in relation to conventional PTC prognostic predictors and Ki-67 and Tg expression. We found that LNR ≥ 21% has a prognostic impact indicating an increased risk of early dissemination with a hazard ratio of 15 compared to patients with LNR < 21%. These findings support the idea that the extent of lymph node metastases might encompass the malignant behavior and risk estimation of PTC better than the anatomical lymph node location^14,44,48,49^.

## Conclusions

There is a heterogeneity of Tg expression and Ki-67 index between the primary PTC and paired lymph node metastases. The Tg expression is inversely correlated to the Ki-67 index. Low expression of Tg in lymph node metastases and LNR ≥ 21% are associated with shorter recurrence-free survival constituting an additional independent predictor to previously known markers such as the N stage. Our data support a potential value of Tg expression determined in lymph node metastases as a complementary factor in predicting the therapeutic impact of radioiodine therapy.

## Data Availability

The data generated and/or analysed during the current study are available from the corresponding author on reasonable request.
